# Examination of the Pupils.—II

**Published:** 1909-04-17

**Authors:** 


### EXAMINATION OF THE PUPILS.—II.

*Inequality of the pupils.*—The next point to observe is whether or not the two pupils are of the same size. In making this observation it is very important indeed that the patient's face should be symmetrically placed as regards the light. Should there be inequality, its degree should be noted as accurately as possible, and here again measurements of the pupils made in the way already described are very desirable. Besides the degree of inequality its persistence or the reverse at successive examinations may also be important.

Slight differences in size between the two pupils are common in perfectly healthy persons. Indeed, it has even been stated that it is rare to find the two of absolutely the same diameter. The natural difference, however, is, as a rule, too slight to attract attention unless very careful investigations are being made. It is true that now and again one sees individuals in whom a more marked difference in the sizes of the two pupils appears to be constant, but as a rule any considerable difference between the two is a symptom of pathological significance.

There are two nerves controlling the movements of the iris, the third cranial nerve and the cervical sympathetic. Excitation of the former causes the pupil to contract; of the latter to dilate. Reversely paralysis of the third cranial nerve leads to dilatation of the pupil, whilst paralysis of the cervical sympathetic results in the pupil of the same side being unduly small. If, therefore, one pupil is markedly smaller than the right, the first thing to determine is which of the two is the abnormal one; this will probably be decided by watching the reactions of each to light and to accommodation, the more mobile pupil being the normal one. If it turns out that the smaller pupil is the abnormal one, the cause of its smallness may be either paralysis of the cervical sympathetic or irritation of the third cranial nerve. Now the effects of an irritant lesion vary, as a rule, from hour to hour and day to day, whereas those of a paralytic lesion are more constant; if, therefore, the degree of inequality of the pupils is one that varies considerably within short spaces of time, irritation is more likely than paralysis, and this is often of considerable help in arriving at a diagnosis.

The four main possibilities are:—

(1) That the left pupil is larger than the right and is the abnormal one: this may be due to paralysis of the pupillary fibres of the left third cranial nerve or to irritation of the left cervical sympathetic. If the third nerve is partly paralysed, it is unlikely that the pupillary fibres alone will be affected, and one would expect paresis of the ciliary muscle or of one or other of the external muscles of the eye-ball. If the cervical sympathetic is irritated, not only will the pupil of that side be enlarged, but there will also be a tendency to undue flushing, heat, and perspiration on that side of the face and possibly a slight widening of the palpebral fissure.

(2) That the left pupil is smaller than the right and is the abnormal one: this may be due to irritation of the left third cranial nerve or to paralysis of the left cervical sympathetic. In the latter case there will be not increased but defective blushing and defective sweating power on the left side of the face, and ptosis of the left upper eyelid.

(3) That the right pupil is the larger and is the abnormal one: the appearances will be similar to those of (2), but the causes will be transposed—that is to say, the condition will be caused by paralysis of the pupillary fibres of the right third cranial nerve or by irritation of the right cervical sympathetic.

(4) That the right pupil is the smaller and is the abnormal one: the cause may be either irritation of the right third cranial nerve or paralysis of the right cervical sympathetic. In the latter case the patient will also have ptosis and defective sweating and blushing power on the right side.

As regards the causes of the irritations or paralyses, as a rule the same lesion is responsible for irritation in an earlier stage and for paralysis in a later. For instance, an aortic aneurysm may be interfering with the left cervical sympathetic; if the case is watched carefully for a long period, it may be noted that, whereas at first the left pupil is constantly larger than the right, later on it is as constantly the smaller of the two. At first the cervical sympathetic is being irritated by the aneurysm; as time goes on the pressure upon the nerve destroys it entirely, allowing uncontrolled action of the third nerve.

Many things may interfere with the cervical sympathetic and cause unequal pupils. It must suffice to enumerate the chief of them—namely, aortic aneurysm; innominate aneurysm; apical fibrosis of lung and pleura; enlargement of the lowest cervical or upper mediastinal glands from inflammation, tubercle, carcinoma secondary to tongue, pharynx, œsophagus, or larynx; sarcoma or Hodgkin's disease; deep scars in the neck; blows; fractures of the first rib; an accessory rib; mediastinal tumours; caries of upper dorsal vertebræ; secondary deposits of growth in the vertebræ; pachymeningitis of the cilio-spinal region of the cord; and degenerative lesions in the grey matter of the cord itself, allied to progressive muscular atrophy or amyotrophic lateral sclerosis. In addition it must be remembered that reflex dilatation of the pupil of the same side readily follows painful stimulation of the skin of the neck by pinching, and precisely similar dilatation may be caused by painful lesions, such as acute cervical adenitis from pediculosis capitis, acute earache, from mastoiditis, or even from a severe boil upon the neck.

A unilateral lesion of the third nerve or of its nucleus is due more often to syphilis than to any other single cause. Rarer affections are direct implication of the nerve in an orbital or intracranial growth, a tumour other than a gumma of the nerve itself, or pressure of a small aneurysm. None of these, however, are such common causes of inequality of the pupils as are vascular conditions which interfere with the function but not with the structure of the nerve. A very well known example of this is traumatic compression of one side of the brain. At first the pupil upon the same side as the injury contracts, the other remaining normal; in an hour or two the pupil upon the sound side begins to contract too, whilst that on the affected side begins to dilate again until a stage of equal pupils is reached once more; in another hour or two the pupil upon the injured side is widely dilated, whilst that on the healthy side is contracted. Finally a stage is reached in which the pupil of the sound side enlarges too, and both pupils are widely and equally dilated It depends, therefore, upon the length of time that has elapsed since the sudden traumatic compression of one side of the brain occurred what the state of the pupils will be.

Compression of one side of the brain which has come on gradually, so that there has been ample time for readjustment of the blood supply to the two sides, does not lead to inequality of the pupils. Any sudden interference with the blood supply of one side may cause pupillary changes similar to those observed in cases of traumatic compression—for instance, cerebral hæmorrhage or cerebral embolism.

Finally, in some elderly patients there is such a degree of atheroma of the vessels of the circle of Willis, and such an unequal distribution of this atheroma upon the two sides, that there is a permanent lack of symmetry in the cerebral circulation and a permanent inequality in the pupils.

